# Effectiveness of the Gaze Direction Recognition Task for Chronic Neck Pain and Cervical Range of Motion: A Randomized Controlled Pilot Study

**DOI:** 10.1155/2012/570387

**Published:** 2012-05-07

**Authors:** Satoshi Nobusako, Atsushi Matsuo, Shu Morioka

**Affiliations:** ^1^Department of Neurorehabilitation, Graduate School of Health Sciences, Kio University, 4-2-2 Umami-naka, Koryo-cho, Kitakatsuragi-gun, Nara 635-0832, Japan; ^2^Department of Rehabilitation, Higashi Osaka Yamaji Hospital, 1-7-5 Inaba, Osaka, Higashiosaka 578-0925, Japan; ^3^Department of Physical Therapy, Faculty of Health Science, Kio University, 4-2-2 Umami-naka, Koryo-cho, Kitakatsuragi-gun, Nara 635-0832, Japan

## Abstract

We developed a mental task with gaze direction recognition (GDR) by which subjects observed neck rotation of another individual from behind and attempted to recognize the direction of gaze. A randomized controlled trial was performed in test (*n* = 9) and control (*n* = 8) groups of subjects with chronic neck pain undergoing physical therapy either with or without the GDR task carried out over 12 sessions during a three-week period. Primary outcome measures were defined as the active range of motion and pain on rotation of the neck. Secondary outcome measures were reaction time (RT) and response accuracy in the GDR task group. ANOVA indicated a main effect for task session and group, and interaction of session. Post hoc testing showed that the GDR task group exhibited a significant simple main effect upon session, and significant sequential improvement of neck motion and relief of neck pain. Rapid effectiveness was significant in both groups. The GDR task group had a significant session-to-session reduction of RTs in correct responses. In conclusion, the GDR task we developed provides a promising rehabilitation measure for chronic neck pain.

## 1. Introduction

 Chronic neck pain lasts longer than six months and is caused by cervical spondylosis deformans, cervical intervertebral disc displacement, or cervical sprain. Conventional therapies include thermotherapy, electric stimulation, and cervical traction [1] and involve soft tissues/joints in the neck in order to alter viscoelastic properties of relevant muscles [2], increase blood flow [3], and separate facet joints [4]. These modalities have been evaluated [5–7], but their efficacies remain unclear [8].

There is increasing evidence that chronic pain problems are characterised by alterations in brain structure and function [9]. The pathological mechanism underlying the prolongation of peripheral pain is thought to involve conflict between sensory-motor cortical processing networks [10, 11]. A cortical model of long-term pain implicated the neural consequences of incongruence between sensory and visuomotor feedback, or prolonged visuosensory-motor conflict [11]. Therapeutic measures such as mirror therapy, motor imagery programming, and virtual visual feedback aim to overcome sensory-motor incongruence and alleviate chronic limb pain. Phantom limb pain was hypothesized to be due to conflict between motor intension and visual-sensory experience in the central nervous system [12]. McCabe et al. examined mirror visual feedback for the treatment of complex regional pain syndrome type 1 and reported reduced limb pain when initiated as early as eight weeks after disease onset [13]. Moseley et al. hypothesized that preceding mirror therapy with the activation of cortical networks without limb movement would reduce pain and swelling and introduced graded motor imagery to reduce chronic limb pain and disability in patients with complex regional pain syndrome type 1 and phantom limb pain [14, 15]. Other studies indicated that visual illusion reduced neuropathic pain in paraplegia [16] and that virtual visual feedback, and simulated motion, alleviated both phantom limb pain [17] and complex regional pain syndrome [18].

A number of mirror therapy pilot studies have addressed phantom limb pain [19] and complex regional pain syndrome [20, 21]. Promising treatment avenues to alleviate chronic pain associated with phantom limb and complex regional pain syndrome include mirror therapy, graded motor imagery, and training with virtual visual feedback, based on the notion that chronic pain is caused by conflict between visual feedback and proprioceptive representation of the damaged limb and that mental practice modalities improve sensory-motor incongruence to activate cortical networks that subserve the affected limb. These measures were effective for chronic limb pain but not neck pain.

Neck motion plays an important role in frequent alterations of gaze direction and is normally achieved by coordinated eye and neck movement. According to the concept of sensory-motor conflict, prolonged neck pain can be attributed to incongruence between sensory and motor feedback in the neck, and to visual-motor conflict in the relevant cortical network areas generated by gaze direction. It is thus possible that a task to overcome sensory-motor incongruence on the alteration of visual direction may alleviate chronic neck pain. Here, we developed a gaze direction recognition (GDR) task in which the subject observed rotation of the neck made by another individual and attempted to recognize gaze direction with reference to neck motion.

## 2. Methods

### 2.1. Study Design and Subjects

**Figure 1:**
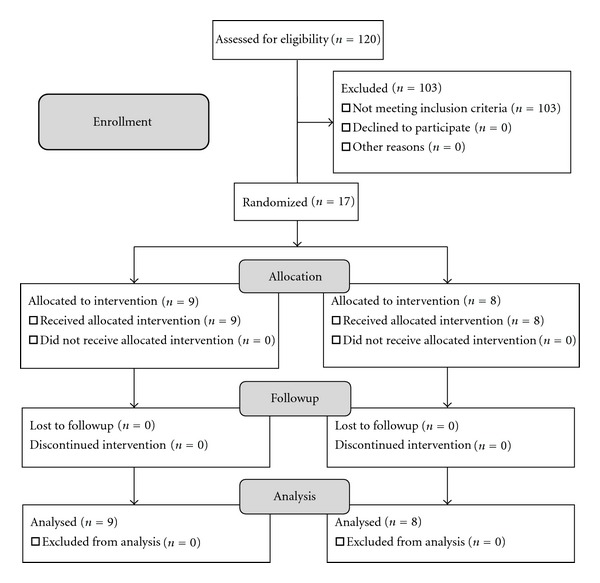
Randomization and allocation of subjects and experimental protocol.

A pilot randomized controlled study was designed to test whether a newly developed gaze direction recognition task could be of potential interest in the treatment of chronic neck pain. One hundred and twenty patients were recruited over a one-week period (March 7 to March 13, 2011) from the Outpatient Department at the Department of Rehabilitation, Higashi-Osaka Yamaji Hospital, and the Midori Clinic (Osaka, Japan). Inclusion criteria were motility disorder in the neck of more than six months' duration with chronic pain and limited range of motion in the neck. Exclusion criteria included cervical or systemic inflammatory signs, and history of surgery in the neck, neural blockage therapy, exercise therapy in the neck, and medications for neck symptoms. According to these criteria, 103 of the initial 120 patient cohort were excluded, and the remaining 17 patients participated as test subjects. Written informed consent, in accordance with the guidelines of the Declaration of Helsinki, was obtained from the 17 subjects prior to the first experimental session.  Figure 1 shows a schematic explanation of enrollment and allocation of subjects, followup, and data analysis.  Table 1 summarizes sex, age, disease and duration, physical therapy given, and the active range of motion and the degree of pain upon neck rotation in each subject.

All 17 subjects were allocated to two groups according to a computer-generated random number. The gaze direction recognition task group (GDR task group, *n* = 9) underwent physical therapy and GDR task sessions as described below. A control group (*n* = 8) received physical therapy but did not undergo the GDR task.

Primary outcome measures included active range of motion and cervical pain, as measured by a 100 mm visual analog scale, upon right and left rotation of the neck. Secondary outcome measures in the GDR task group included reaction time and the accuracy of responses in the GDR task.

A single session involving an interventional procedure was carried out as follows. After a routine physical check-up, carried out by a physician, all subjects were evaluated for the active range of motion and cervical pain upon rotation of the neck. Thereafter, subjects were administered physical therapies. Subsequent to this, the GDR task group, but not the control group, underwent a GDR task. Finally, all subjects in the two groups were assessed for active range of neck motion and evaluated for pain on neck rotation.

A total of 11 interventional sessions were performed over a total period of three weeks. A followup assessment was carried out 15 days after the last session.

Physical therapies were managed by two physical therapists unaware of which subject group they were assessing. The active ranges of neck motion and cervical pain upon rotation of the neck were assessed independently by the experimenter, the experimenter's assistant, and the response recorder, all unaware of which subject group they were assessing.

This study was approved by the ethics committee of the Moujin-kai medical corporation (approval number: H22-12) and Kio University Health Science Graduate School (approval number: H19-12).

### 2.2. Gaze Direction Recognition Task

**Figure 2:**
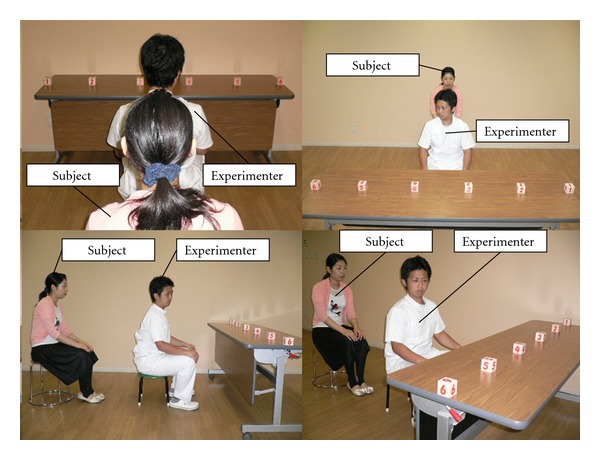
Experimental design of gaze direction recognition task. Each column represents the positional relationship between a subject and an experimenter with six numbered boxes. The subject is positioned behind the experimenter and views neck rotation of the experimenter who attempts to gaze randomly at one of six boxes placed on the table, and imagines which one of the boxes the experimenter directs his gaze upon. The subject was then asked to give a verbal response as to the box number of the experimenter's gaze direction.

We developed a task for gaze direction recognition (GDR) in which the subject was asked to view an experimenter from behind, to observe the rotation of the experimenter's neck associated with the change of gaze direction, and to attempt to recognize the experimenter's direction of gaze (Figure 2). Gaze direction recognition of another individual is evidently related to activity of the superior temporal sulcus of the cerebral cortex [22]. However, when an individual is viewed from behind, with his or her face or eye invisible, the observer has to simulate himself with the other individual's neck rotation towards the direction of gaze in order to recognize the another individual's direction of gaze. This type of motor simulation is eponymous with motor imagery [23]. Being similar to real motion, motor imagery involves activity of the motion-related cortical areas including the premotor area, supplementary motor area, and primary motor area [24]. It has been reported that imagery of voluntary movement of the fingers, toes, and tongue, activates corresponding body-part-specific motion representations in the motor cortex [25]. It has also been suggested that observations of another individual's motion activate the mirror neuron system [26] consisting of the superior temporal sulcus, supramarginal gyrus (inferior parietal lobule, Brodmann area 40), and premotor cortex. Action observation has been demonstrated in a functional MRI study to activate premotor and parietal areas in a somatotopic manner; when individuals observe an action, an internal replica of that action is automatically generated in their premotor cortex [27]. Action observation therapy utilizing the activation of the mirror neuron system provides a positive impact upon rehabilitation of the upper limb motor deficits after stroke [28]. In a previous study, we measured changes of oxygenated hemoglobin (oxyHb) in the cortical blood circulation using functional near-infrared spectroscopy and found that oxyHb concentrations were significantly increased during the GDR task in the premotor area, as well as in the superior temporal sulcus, as compared with those during the action observation of another individual [29]. The GDR task differs from simple action observation in that internally simulated motion of neck rotation is required for the subject together with observation of the another individual's neck rotation. We suggested in our previous study that since a test of simple observation did not activate the premotor area, then it follows that the activated premotor area during the GDR task may represent simulated motion of the neck. We hypothesized, on the basis of our previous study, that the GDR task is crucial in the development of motor imagery and provides potential rehabilitation for chronic neck pain.

### 2.3. Gaze Direction Recognition Task Procedure

The 9 subjects belonging to the GDR task group underwent a specific task following physical therapy (Figure 2). An experimenter sat 75 cm apart from a subject, and the subject was asked to observe the experimenter from behind. A table (1800 mm × 400 mm) was placed 75 cm in front of the experimenter, on which six blocks, numbered 1 to 6, were placed in regular intervals. Subjects were able to watch all of the blocks. The experimenter's gaze changed to either one of the six numbered blocks in a random manner by voluntary eye movement and rotation of the neck. The experimenter initiated the performance following a specific signal by an assistant to the experimenter. The experimenter maintained gaze at a certain numbered box until the subject gave a response. Then, the subject observed the experimenter's neck rotation from behind and was asked to imagine the block at which the experimenter was gazing and to provide a verbal response as to which imagined block the subject was gazing at as quickly as possible. Whether the subject's recognition of the experimenter's gaze of direction was correct was not fed-back to the subject until the end of the experiment. An assistant to the experimenter recorded the reaction times and correctness of the response. A single experimental GDR task consisted of 30 trials of the task outlined above, which was carried out in about 10 minutes.

The subjects were instructed not to move their body during the GDR task. To monitor the subjects' behavior during the GDR task, electromyography (Biometrics Ltd, USA) was recorded from the sternocleidomastoid muscle and analyzed using the TRIAS System (DKH Ltd, Japan).

Subjects of both the GDR task and control group received physical therapies, consisting of either one of three therapeutic modalities: cervical traction (*n* = 17), microwave therapy (*n* = 13), or interferential current (*n* = 3). Physical therapy modality was selected by the physician (Table 1) and performed by physical therapists who were unaware of the allocated group. 

### 2.4. Primary Outcome Measures

Active range of cervical rotation motion was measured using a Goniometer (Q110, Biometrics Ltd) according to the measurement method of active range of motion that was recommended in 1995 by the Japanese Orthopaedic Association and the Japanese Association of Rehabilitation Medicine, based upon methods described by the American Academy of Orthopaedic Surgeons (1965). Measurements were analyzed using the TRIAS System (DKH Ltd). Subjects sat in a chair, and a vertical line connecting the bilateral acromion was defined as the primary reference axis, whilst a line connecting the bridge and occipital tubercle was defined as the rotational reference axis. During measurements of active range of motion upon neck rotation, the assistant sustained the subject's posture in order to maintain the trunk to form the basic axis. Active range of motion in neck rotation to the right and neck rotation to the left was each measured three times, and the third measurement was recorded for analysis.

Neck pain was assessed using the 100 mm visual analog scale (VAS). The subject was asked to mark the horizontal line of the scale according to the strength of pain after he or she rotated the neck to either the right or left. The left end of the scale was defined as no pain and the opposite right end of the scale was defined as maximum. The subject was not informed about previous measurements at the time of posttask measurement.

Active range of motion evaluation and VAS pain assessment were performed before and after each interventional session in both the GDR task group and control group.

Measurements were taken in an examination room by one experimenter, an assistant to the experimenter, and one recorder. The assistant and recorder were not informed of the assignment of subject group.

### 2.5. Secondary Outcome Measures

In the GDR task group, response reaction time and accuracy of GDR task (number of correct answers/total number of answers x 100) were determined. The reaction times between the starting signal by an assistant and the subject's response were measured using a stop-watch at an order of milliseconds. Reaction times for the correct recognition of gaze direction were selected for further analyses.

### 2.6. Statistical Analyses

Statistical analyses were performed using SPSS for windows, and an alpha level of 5% was considered as statistically significant.

Age and disease duration at the first experimental session, and active range of neck motion and VAS pain assessment before intervention were compared between the GDR task group and the control group using the unpaired *t*-test. Sex, disease entity, and physical therapy were compared between the GDR group and the control group using the chi-squared test.

The outcome measures of the active range of motion and VAS pain assessment upon lateral neck rotation were analyzed using two-way ANOVA for two binary factors, that is, group (GDR task group and control group) and task session (12 sessions). The Bonferroni method was used for post hoc testing.

In order to analyze the rapid efficacy of intervention, active range of motion and VAS pain assessment before and after intervention were compared between the GDR group and the control group using a paired *t*-test.

To evaluate the sequential changes of gaze direction recognition ability associated with repetitive GDR task achievement, session-to-session measurements of reaction times for correct recognitions (12 sessions) and accuracy of responses (12 sessions) in the GDR task group were statistically analyzed using one-way ANOVA and the Bonferroni method with post hoc testing.

In the GDR task group, correlations between reactions times and correct recognitions, accuracy of responses, active range of motion, and VAS pain assessment in cervical rotations, were determined using the Pearson correlation coefficient.

## 3. Results

None of the 17 subjects in either the GDR group or the control group withdrew from the study (Figure 1). Electromyographies in the bilateral sternocleidomastoid muscles demonstrated that all subjects of the GDR task group remained stable in the cervical muscles during the GDR task sessions.

### 3.1. Baseline Data

Table 1 shows patient sex, age, disease, disease duration, type of physical therapies given, and active range of motion and VAS pain assessment on neck rotation before interventions. The unpaired *t*-test revealed no significant difference between the GDR task group and the control group in terms of age and disease duration at the time of the first interventional session (age: 95% confidence interval (CI) 11.5 to −19.6, *P* = 0.587; disease duration in days: 95% CI 64.7 to −60.4, *P* = 0.942; right rotation aROM: 95% CI 0.5 to −16.8, *P* = 0.063; left rotation aROM: 95% CI 8.5 to −5.5, *P* = 0.657; right rotation pain VAS: 95% CI 35.9 to −6.0, *P* = 0.148; left rotation pain VAS: 95% CI 12.8 to −24.3, *P* = 0.517). Chi-squared tests for sex, disease, and physical therapy showed no significant difference between the GDR task group and control group (sex: *χ* ^2^ = 0.0525, *P* = 0.9741; disease: *χ* ^2^ = 1.2364, *P* = 0.5389; physical therapy (physiotherapy): *χ* ^2^ = 0.4392, *P* = 0.8028).

**Figure 3:**
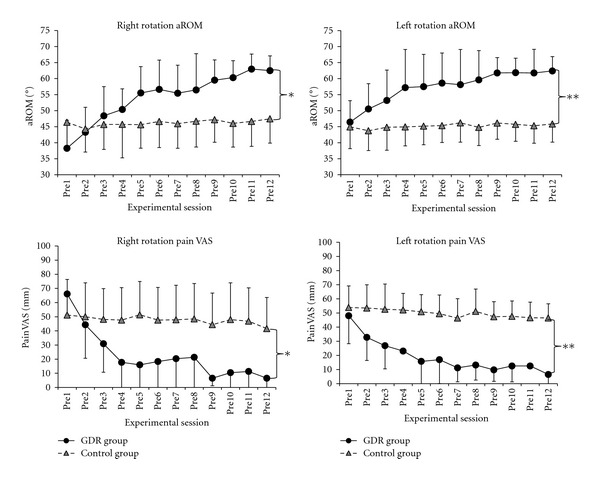
Sequential data of active range of motion and pain assessment in the gaze direction recognition task group and control group. Active range of motion (in degrees) and pain visual analog scale (in mm) obtained before each of 12 sessions in the gaze direction recognition task group (*n* = 9) and control group (*n* = 8). Data points represent mean of the relevant group and bars standard deviation. Left columns: right rotation of the neck; right columns: left rotation of the neck. Two-way repeated-measures ANOVA analysis revealed a main effect of group, *P < 0.05; **P < 0.01. Interaction effect between the group and session is significant in all data of both groups. A main effect of session was significant only in the GDR task group (*P* < 0.05).

**Figure 4:**
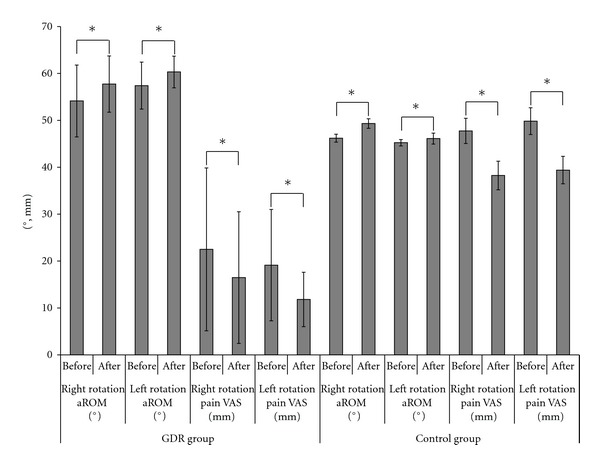
Active range of motion and pain assessment before and after gaze direction recognition in the task group and control group. *paired *t*-test, *P* < 0.01.

Table 2 and Figure 3 show sequential changes and statistical analyses of active range of motion and pain VAS when subjects rotated their necks to the right or left.  Figure 4 shows the active range of motion and pain assessment before and after the task, along with associated statistical analysis. 

### 3.2. Active Range of Motion on Neck Rotation to the Right

Statistical analyses using two-way repeated-measures ANOVA showed a significant main effect of group, *F*(1, 15) = 6.177, *P* = 0.025, a main effect of interventional session, *F*(4.797, 71.952) = 14.335, *P* = 0.000000002, and a significant interaction effect between group and interventional session, *F*(4.797, 71.952) = 11.051, *P* = 0.0000001. The GDR task group had a significant main effect of interventional session, GDR task group  *F*(11, 5) = 27.768, *P* = 0.001; Control group *F*(11, 5) = 0.180, *P* = 0.992. Post hoc tests indicated significant sequential improvement in the GDR task group, but not in the control group (Table 2, Figure 3).

As regarding rapid effectiveness, both the GDR group and control group showed a significant improvement, GDR task group: 95% CI −2.8 to −4.4, *P* = 0.00000000000000611; control group: 95% CI −1.7 to −3.7, *P* = 0.00000027438 (Figure 4).

### 3.3. Active Range of Motion on Neck Rotation to the Left

Statistical analyses using two-way repeated-measures ANOVA showed a significant main effect of group, *F*(1, 15) = 15.059, *P* = 0.001, a main effect of interventional session, *F*(3.616, 54.237) = 9.429, *P* = 0.00001, and a significant interaction effect between group and interventional session, *F*(3.616, 54.237) = 6.626, *P* = 0.0003. The GDR task group had a significant main effect of interventional session, GDR task group *F*(11, 5) = 18.697, *P* = 0.002; Control group *F*(11, 5) = 1.206, *P* = 0.445. Post hoc tests indicated significant sequential improvement in the GDR task group, but not in the control group (Table 2, Figure 3).

As regards rapid effectiveness, both the GDR group and control group showed a significant improvement, GDR task group: 95% CI −2.2 to −3.6, *P* = 0.00000000000319147; control group: 95% CI −0.3 to −1.1, *P* = 0.001613966 (Figure 4).

### 3.4. VAS Pain Assessment upon Right Rotation of the Neck

Statistical analyses using two-way repeated-measures ANOVA showed a significant main effect of group, *F*(1, 15) = 7.398, *P* = 0.016, a main effect of interventional session, *F*(3.937, 59.062) = 12.477, *P* = 0.0000002, and a significant interaction effect between group and interventional session, *F*(3.937, 59.062) = 8.374, *P* = 0.00002. The GDR task group had a significant main effect of interventional session, GDR task group  *F*(11, 5) = 18.601, *P* = 0.002; Control group *F*  (11, 5) = 1.318, *P* = 0.403. Post hoc tests indicated significant sequential improvement in the GDR task group, but not in the control group (Table 2, Figure 3).

As regards rapid effectiveness, both the GDR group and control group showed a significant improvement, GDR task group: 95% CI 8.1 to 4.0, *P* = 0.00000006802114357; control group: 95% CI 10.4 to 6.7, *P* = 0.00000000000001 (Figure 4).

### 3.5. VAS Pain Assessment upon Left Rotation of the Neck

Statistical analyses using two-way repeated-measures ANOVA showed a significant main effect of group, *F*(1, 15) = 27.183, *P* = 0.0001, a main effect of interventional session, *F*(3.757, 56.356) = 8.143, *P* = 0.00004, and a significant interaction effect between group and interventional session, *F*(3.757, 56.356) = 3.614, *P* = 0.012. The GDR task group had a significant main effect of interventional session, GDR task group *F*(11, 5) = 4.945, *P* = 0.045; Control group *F*(11, 5) = 0.423, *P* = 0.891. Post hoc tests indicated significant sequential improvement in the GDR task group, but not in the control group (Table 2, Figure 3).

As regards rapid effectiveness, both the GDR group and control group showed a significant improvement, GDR task group: 95% CI 9.8 to 4.8, *P* = 0.00000005298285533; control group: 95% CI 11.8 to 6.9, *P* = 0.00000000001 (Figure 4).

### 3.6. Reaction Times in Correct Recognition and Accuracy of Responses in the GDR Task

Table 3 shows session-to-session sequential changes in reaction times for correct answers and the accuracy of responses in the GDR task group. One-way ANOVA revealed that the reaction times for correct answers exhibited a significant reduction decrease session 1 and session 10 (95% CI 1150.7 to 40.6, one-way ANOVA, *P* = 0.021) and between session 1 and session 12 (95% CI 1156.0 to 45.9, one-way ANOVA, *P* = 0.019). There was no significant sequential change in the accuracy of responses.

### 3.7. Correlation Analyses

**Figure 5:**
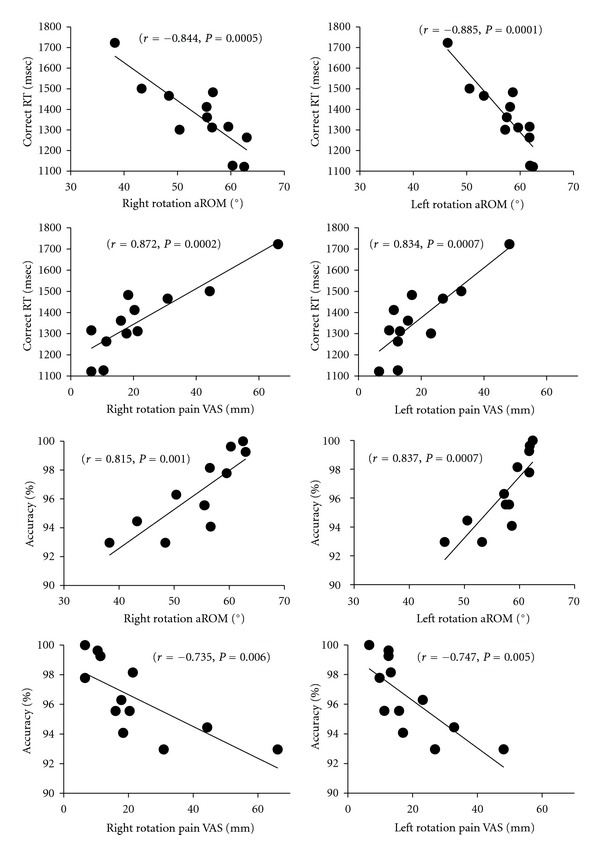
Correlation data between rotation of the neck and reaction times for correct answers and those between pain assessment and accuracy of responses in the GDR task group. Left columns: right rotation of the neck; right columns: left rotation of the neck. The data indicate significant correlations in all (*P* < 0.01).

Figure 5 illustrates reaction times for correct answers versus the active range of motion upon neck rotations, reaction times for correct answers versus pain assessment upon neck rotations, accuracy of responses versus active range of motion upon neck rotations, and the accuracy of responses versus pain assessment upon neck rotations.

**Figure 6:**
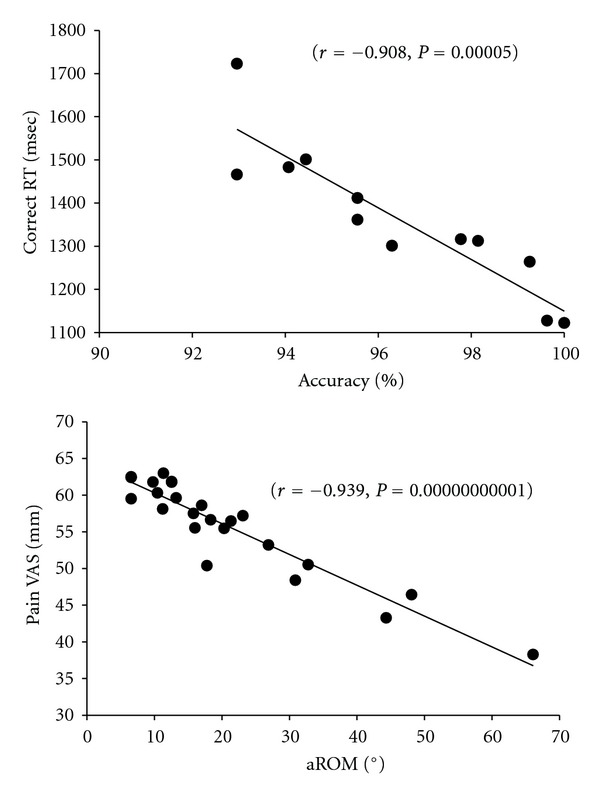
Correlation data between the accuracy of response and the reaction times for correct answers, and correlative data between active range of motion and pain assessment of the neck. Data indicate significant correlations in all parameters (*P* < 0.01).

Figure 6 shows reactions times for correct answers versus the accuracy of responses, and the active range of motion versus pain assessment upon neck rotation. These data were obtained from the GDR task group, and all relationships indicate significant correlations (*P* < 0.01).

## 4. Discussion

Results from the GDR task revealed significant sequential relief of chronic neck pain as assessed by the visual analog scale (VAS). Furthermore, an intergroup statistical comparison indicated that the improvement of VAS pain assessment was significant in the GDR task group as compared with the control group without the GDR task. It has been hypothesized that chronic pain is due to incongruence between sensory feedback signals and visuosomatic feedback signals in the relevant cortical areas and that prolonged conflict results in long-term plasticity in the relevant cortex which leads to difficulties in treatment [10, 11]. There is a fair amount of evidence to support the view that patients with chronic limb pain exhibit delayed reaction times in the hand or foot recognition task, a response that requires motor imagery of the responsible organs [30–32]. It is probable that a significant correlation of delayed reaction times of motion with the duration of disease symptoms and chronic pain reflects damage in the intracranial reorganization of the cortical representation of body schema and involves motion programming in the motor cortex [30]. Patients with complex regional pain syndrome, phantom pain, and chronic low back pain, are shown to be associated with altered central neural processing in the somatosensory imagery areas, which may cause shrinkage of representational areas and sensory impairment [33], leading to distorted body image [34, 35]. Reduced cortical activity during motor imagery of the affected limb has been implicated in patients with chronic pain [36]. The pathological mechanism through which cervical motion disorders are accompanied by prolongation of neck pain can be considered as follows. First, organic disorders in the neck such as cervical spondylosis deformans, and cervical intervertebral disc displacement, induce pain and limited range of motion in the neck. Prolonged inhibition of cervical motion may result in compensatory motion to reduce pain. Such compensatory muscle activities may trigger painful spasms of the neck muscles. This viscous circle strengthens compensatory motion and alters motor programming in cortical motor processing. Once the underlying organic disorders are resolved, appropriate revision of motor program in the cortex is likely to relieve pain. Moseley reported that the hand laterality recognition task reduced pain and disability together with reduced reaction times in the task for patients with chronic hand pain [14, 15]. Similar beneficial effects are likely to be the case for our current results in that the GDR task produced a sequential reduction in reaction times for correct recognition of another individual's direction of gaze and significant correlates of reduced reaction times and accuracy of responses were associated with improvement of chronic neck pain. Action observations of the experimenter's neck rotations promptly affected subjects to imagine the direction of gaze and to induce precise motor imagery of the neck [25]. Action observations of neck rotations in a healthy experimenter without neck disease may produce neural motor images and activate neck-specific-motor representations in the cortex [27]. 

Our control group showed rapid improvement in the active ranges of motion and pain in neck rotation, although this improvement did not remain long. A previous study on the effects of cervical traction reported subjective relief of neck pain as late as 12 hours after intervention [37], suggesting that traction therapy provides rapid effectiveness. However, physical therapies provide only short-term improvement in neck pain and active range of motion, and such treatment modalities are not sufficient to achieve frequently performed cervical motion in daily life according to the cervical motion program after neck damage. In the GDR task group, programming for precise cervical motion was facilitated together with rapid peripheral effectiveness by physical therapies as in the control group, which must have been responsible for the persistent effectiveness revealed by followup examination 15 days after intervention in the GDR task group.

Sequential changes in the GDR task group also included significant improvement in the active range of neck rotation motion, although the control group of diseased subjects without the GDR task did not show such sequential improvement. Furthermore, in a comparison between the groups, the GDR task group revealed significant improvement in the active motion range of neck lateral rotations. The GDR task is responsible for a type of motor imagery. Motor imagery increases muscle contractions [38], enhances body balance in elderly women [39], increases precision of skill and improves motion timing [40], and alleviates poststroke hemiparesis [41]. In the present study, we determined a significant correlation between reduced reaction times and the enhancement of response accuracy in the GDR task group with improvement of active range of neck motion. However, limited information is available concerning the sequential improvement of active range of motion as a beneficial product of motor imagery [42]. Sequential relief of chronic pain and a negative correlation between VAS pain assessment and active range of motion were observed in the current study, suggesting that reduced pain may be related to improvement of the active range of motion.

Limitations of this pilot study include a small size of sample subjects. Reaction times and response accuracy in the GDR task in healthy individuals and an additional larger number of patients with chronic cervical pain were, however, justified. Cortical areas and neural networks relevant to the GDR task remain to be elucidated using refined research technologies including neuroimaging techniques. Ages of our subjects varied greatly from 16 to 74 years, although there was no significant difference in age between the two groups. Nevertheless, it must be appreciated that in general, active range of motion is thought to be related to age and that the pathogenesis of neck pain can be quite different between the young and old. It is therefore possible that chronic neck pain or cervical range of motion was improved by alternative mechanisms acting within each age group. Consequently, there is a need for future research to explore whether GDR efficacy differs between different age groups. Primary outcome measurement was limited to neck pain and cervical range of motion since the subjects were ambulatory outpatients without problems associated with activities of daily living (ADL). Another reason for avoiding ADL evaluation was that we performed a pilot experiment to explore whether or not GDR was efficacious for neck pain treatment. However, since the present study demonstrated the pain alleviation effect of GDR, the next step would be to examine if GDR influences ADL and the quality of life in patients with neck pain. Unfortunately, we did not evaluate psychophysiological aspects of GDR efficacy in the present study. It was reported that chronic pain is perpetuated or aggravated by several factors, including psychological measures such as passive mental processes or thinking and catastrophizing [43]. We suggest that GDR efficacy should be analyzed in more detail in the future by considering the differential effects of psychophysiological factors upon GDR efficacy. In the present study, we did not evaluate coordinated movement of the eye and neck, activity of neck muscles, or neck proprioception. We consider that such studies in the future will yield novel findings concerning the mechanisms of chronic pain occurring in patients with neck motility disorders. This will contribute enormously to the development of therapeutic interventions for chronic pain. We followed up our subjects for only a very short period of 15 days following the last session. It is also necessary to extend the intervention, as well asthe length of followup period, in order to verify our GDR efficacy results. Moreover, it is important to investigate GDR efficacy in patients who have suffered from a motility disorder of the neck for a shorter period than six months to assess whether earlier GDR intervention is effective. 

## 5. Conclusions

A randomized clinical trial to study the effects of a gaze direction recognition task upon cervical rotation and pain in patients with chronic neck pain revealed that a sequence of tasks effectively improves active range of neck rotation and reduces pain. Results suggest that the gaze direction recognition task provides a potential therapeutic measure for the treatment of chronic neck pain.

## Figures and Tables

**Table 1:** Patient sex, age, disease, duration of disease, physical therapy, aROM and pain VAS before the first intervention.

Sex (M/F)	Age (years)	Disease	Duration (days)	Physiotherapy	Right aROM (°)	Left aROM (°)	Right pain VAS (mm)	Left pain VAS (mm)
F	65	Cervical spondylosis	372	Cervical traction and microwave therapy	40.2	50.5	72	44
M	47	Cervical sprain	249	Cervical traction	40.7	40.2	83	76
F	16	Cervical sprain	261	Cervical traction and microwave therapy	45.2	60.2	68	43
M	61	Cervical spondylosis	269	Cervical traction and microwave therapy	30.3	50.1	63	46
M	52	Cervical spondylosis	198	Cervical traction and microwave therapy	42.3	46.2	53	36
F	55	Cervical spondylosis	272	Cervical traction and microwave therapy	50.4	38.4	42	58
F	74	Cervical spondylosis	207	Cervical traction and interferential current	35.2	40.6	62	66
M	32	Cervical sprain	311	Cervical traction and microwave therapy	20.6	45.4	90	7
M	51	Cervical spondylosis	269	Cervical traction and microwave therapy	39.5	46.2	62	57
GDR group mean (SD)	50.3 (17.5)		267.6 (52.1)		38.3 (8.7)	46.4 (6.7)	66.1 (14.5)	48.1 (19.9)
F	35	Cervical sprain	216	Cervical traction and microwave therapy	44.2	55.4	69	23
M	65	Cervical spondylosis	232	Cervical traction and microwave therapy	40.3	44.1	66	54
M	70	Cervical spondylosis	239	Cervical traction and microwave therapy	52.3	42.3	1	52
M	43	Cervical sprain	198	Cervical traction and microwave therapy	30.4	38.1	78	65
F	61	Cervicobrachial syndrome	392	Cervical traction and interferential current	54.2	36.3	28	72
F	52	Cervical spondylosis	337	Cervical traction and interferential current	48.4	41.6	63	66
F	51	Cervical spondylosis	217	Cervical traction and microwave therapy	50.2	49.3	52	45
M	58	Cervical spondylosis	292	Cervical traction and microwave therapy	51.1	52.4	52	54
Control group mean (SD)	54.4 (11.6)		265.4 (68.7)		46.4 (7.9)	44.9 (6.8)	51.1 (25.2)	53.9 (15.3)

Arom: active range of motion; VAS: visual analog scale for pain assessment. Right aROM: active range of motion of rotation of the neck to the right before the first intervention; left aROM: active range of motion of rotation of the neck to the left before the first intervention; right pain VAS: pain visual analog scale on right rotation of the neck before the first intervention; left pain VAS: pain visual analog scale on left rotation of the neck before the first intervention. GDR group, mean (standard deviation); control group, mean (standard deviation). In each variable, there was no significant difference between the two groups.

**Table 2:** aROM and pain VAS in the GDR task group and control group, measured before each experimental session.

				Experimental session																					
			Session 1	Session 2		Session 3		Session 4		Session 5		Session 6		Session 7		Session 8		Session 9		Session 10		Session 11		Session 12	
GDR group (*n* =9)	Right rotation aROM (°)	Pre	38.3	43.3		48.4		50.4	*1	55.5	**1 *2	56.6	**1 *2	55.5	**1 **2 *3	56.5	**1 *2 *3	59.5	**1 **2 **3 *4	60.3	**1 **2 **3 **4	63.0	**1 **2 **3 **4 *7	62.5	∗∗1 ∗∗2 ∗∗3 ∗∗4
		SD	8.7	7.8		9.1		6.4		8.2		9.1		8.7		11.3		6.3		5.3		4.7		4.6	

	Left rotation aROM (°)	Pre	46.4	50.5		53.2		57.2	*1	57.5	**1	58.6	**1 **2 **3	58.1	**1 *3	59.6	**1 **2	61.8	**1 **2 **3	61.8	**1 **2 *3	61.8	**1 **2	62.4	**1 **2 *3
																								4.5	
		SD	6.7	7.9		9.5		11.9		10.1		9.4		11.0		9.1		4.8		4.5		7.4			
	Right rotation pain VAS (mm)	Pre	66.1	44.3	**1	30.9	**1	17.8	**1	16.0	**1 *2	18.3	**1	20.3	**1	21.3	**1	6.6		10.4		11.3	**1 **2	6.6	
																			**1 **2 **3		**1 **2 *3				**1 **2 **3
		SD	14.5	23.7		20.1		26.1		19.5		30.8		25.1		22.8		5.3		12.1		18.2		8.5	
	Left rotation pain VAS (mm)	Pre	48.1	32.8		26.9	**1	23.1		15.8	**1	17.0	**1	11.2	**1 **2	13.2	**1 **2	9.8	**1 **2	12.6	**1	12.6	**1	6.6	
																									**1 **2 **3
		SD	19.9	16.3		16.4		32.5		16.6		17.0		9.9		10.7		8.1		11.3		24.1		8.6	

Control group (*n* = 8)	Right rotation aROM (°)	Pre	46.4	44.3		45.7		45.7		45.6		46.6		46.0		46.7		47.2		46.0		46.7		47.5	
		SD	7.9	7.2		7.8		10.4		7.3		8.2		7.7		8.0		7.1		7.4		7.8		7.6	
	left rotation aROM (°)	Pre	44.9	43.7		44.8		44.9		45.2		45.3		46.2		44.8		46.1		45.7		45.3		45.8	
		SD	6.8	6.2		7.2		5.9		5.8		5.3		6.0		5.7		5.1		5.3		5.5		5.7	
	Right rotation pain VAS (mm)	Pre	51.1	50.0		48.1		47.6		51.4		47.6		47.9		48.5		44.4		48.0		46.9		41.6	
		SD	25.2	24.0		21.8		22.9		23.6		23.1		24.4		25.0		22.4		26.0		23.6		22.0	
	Left rotation pain VAS (mm)	Pre	53.9	53.5		52.6		52.1		50.9		49.5		46.4		51.1		47.4		47.6		46.6		46.5	
		SD	15.3	16.5		17.9		11.8		12.1		13.3		13.7		15.8		10.6		10.9		11.1		10.1	

Arom: active range of motion; VAS: visual analog scale for pain assessment. Pre: average in measurements before task; SD: standard deviation in measurements. *Significance in two-way repeated-measures ANOVA and factorial analysis of session by Bonferroni ad hoc test. *: < 0.05; **: < 0.01. The numbers after * represent the number of task session. In all parameters, the GDR task group showed significant sequential improvement, while the control group did not.

**Table 3:** Sequential reaction time data for the correct recognition of the experimenter's direction of gaze and response accuracy in the GDR task group.

	Experimental session											
	Session 1	Session 2	Session 3	Session 4	Session 5	Session 6	Session 7	Session 8	Session 9	Session 10	Session 11	Session 12
Correct RT (msec)	1722.9	1501.0	1465.9	1300.9	1361.3	1482.8	1411.6	1312.2	1316.0	1127.2*	1263.8	1121.9*
SD	558.7	446.8	345.4	234.9	380.9	464.1	287.2	280.8	190.9	130.6	323.3	90.7
Accuracy (%)	93.0	94.4	93.0	96.3	95.6	94.1	95.6	98.1	97.8	99.6	99.3	100.0
SD	6.1	6.5	8.6	5.4	5.3	7.0	7.5	4.4	4.7	1.1	2.2	0.0

Correct RT **=** reaction times in correct recognition. Accuracy **=** response accuracy in the GDR task group. Sequential data from session 1 to session 12; mean and standard deviation (*n* = 9). *one-way ANOVA (*P* < 0.05).

## References

[1] Rush PJ, Shore A (1994). Physician perceptions of the value of physical modalities in the treatment of musculoskeletal disease. *British Journal of Rheumatology*.

[2] Lehmann JF, Masock AJ, Warren CG, Koblanski JN (1970). Effect of therapeutic temperatures on tendon extensibility. *Archives of Physical Medicine and Rehabilitation*.

[3] Wessman HC, Kottke FJ (1967). The effect of indirect heating on peripheral blood flow, pulse rate, blood pressure, and temperature. *Archives of Physical Medicine and Rehabilitation*.

[4] Judovich B, Nobel GR (1957). Traction therapy, a study of resistance forces: preliminary report on a new method of lumbar traction. *The American Journal of Surgery*.

[5] Borman P, Keskin D, Ekici B, Bodur H (2008). The efficacy of intermittent cervical traction in patents with chronic neck pain. *Clinical Rheumatology*.

[6] Young IA, Michener LA, Cleland JA, Aguilera AJ, Snyder AR (2009). Manual therapy, exercise, and traction for patients with cervical radiculopathy: a randomized clinical trial. *Physical Therapy*.

[7] Chiu TTW, Ng JK-F, Walther-Zhang B, Lin RJH, Ortelli L, Chua SK (2011). A randomized controlled trial on the efficacy of intermittent cervical traction for patients with chronic neck pain. *Clinical Rehabilitation*.

[8] Chou R, Qaseem A, Snow V (2007). Diagnosis and treatment of low back pain: a joint clinical practice guideline from the American College of Physicians and the American Pain Society. *Annals of Internal Medicine*.

[9] Wand BM, Parkitny L, O’Connell NE (2011). Cortical changes in chronic low back pain: current state of the art and implications for clinical practice. *Manual Therapy*.

[10] Harris AJ (1999). Cortical origin of pathological pain. *The Lancet*.

[11] McCabe CS, Haigh RC, Halligan PW, Blake DR (2005). Simulating sensory-motor incongruence in healthy volunteers: implications for a cortical model of pain. *Rheumatology*.

[12] Ramachandran VS, Rodgers-Ramachandran D (1996). Synaesthesia in phantom limbs induced with mirrors. *Proceedings of the Royal Society B*.

[13] McCabe CS, Haigh RC, Ring EFJ, Halligan PW, Wall PD, Blake DR (2003). A controlled pilot study of the utility of mirror visual feedback in the treatment of complex regional pain syndrome (type 1). *Rheumatology*.

[14] Moseley GL (2004). Graded motor imagery is effective for long-standing complex regional pain syndrome: a randomised controlled trial. *Pain*.

[15] Moseley GL (2006). Graded motor imagery for pathologic pain: a randomized controlled trial. *Neurology*.

[16] Moseley GL (2007). Using visual illusion to reduce at-level neuropathic pain in paraplegia. *Pain*.

[17] Mercier C, Sirigu A (2009). Training with virtual visual feedback to alleviate phantom limb pain. *Neurorehabilitation and Neural Repair*.

[18] Sato K, Fukumori S, Matsusaki T (2010). Nonimmersive virtual reality mirror visual feedback therapy and its application for the treatment of complex regional pain syndrome: an open-label pilot study. *Pain Medicine*.

[19] Chan BL, Witt R, Charrow AP (2007). Mirror therapy for phantom limb pain. *The New England Journal of Medicine*.

[20] Vladimir Tichelaar YIG, Geertzen JHB, Keizer D, Paul van Wilgen C (2007). Mirror box therapy added to cognitive behavioural therapy in three chronic complex regional pain syndrome type I patients: a pilot study. *International Journal of Rehabilitation Research*.

[21] Selles RW, Schreuders TAR, Stam HJ (2008). Mirror therapy in patients with causalgia (complex regional pain syndrome type II) following peripheral nerve injury: two cases. *Journal of Rehabilitation Medicine*.

[22] Hoffman EA, Haxby JV (2000). Distinct representations of eye gaze and identity in the distributed human neural system for face perception. *Nature Neuroscience*.

[23] Jeannerod M (1994). The representing brain: neural correlates of motor intention and imagery. *Behavioral and Brain Sciences*.

[24] Lotze M, Montoya P, Erb M (1999). Activation of cortical and cerebellar motor areas during executed and imagined hand movements: an fMRI study. *Journal of Cognitive Neuroscience*.

[25] Ehrsson HH, Geyer S, Naito E (2003). Imagery of voluntary movement of fingers, toes, and tongue activates corresponding body-part-specific motor representations. *Journal of Neurophysiology*.

[26] Rizzolatti G, Luppino G, Matelli M (1998). The organization of the cortical motor system: new concepts. *Electroencephalography and Clinical Neurophysiology*.

[27] Buccino G, Binkofski F, Fink GR (2001). Action observation activates premotor and parietal areas in a somatotopic manner: an fMRI study. *European Journal of Neuroscience*.

[28] Ertelt D, Small S, Solodkin A (2007). Action observation has a positive impact on rehabilitation of motor deficits after stroke. *NeuroImage*.

[29] Nobusako S, Shimizu S, Miki K, Tamaki H, Morioka S (2010). Neural basis for perception of gaze direction by observation from behind: a study using functional near-infrared spectroscopy. *Rigakuryoho Kagaku*.

[30] Moseley GL (2004). Why do people with complex regional pain syndrome take longer to recognize their affected hand?. *Neurology*.

[31] Coslett HB, Medina J, Kliot D, Burkey AR (2010). Mental motor imagery indexes pain: the hand laterality task. *European Journal of Pain*.

[32] Coslett HB, Medina J, Kliot D, Burkey A (2010). Mental motor imagery and chronic pain: the foot laterality task. *Journal of the International Neuropsychological Society*.

[33] Schwenkreis P, Maier C, Tegenthoff M (2009). Functional imaging of central nervous system involvement in complex regional pain syndrome. *American Journal of Neuroradiology*.

[34] Sumitani M, Yozu A, Tomioka T, Yamada Y, Miyauchi S (2010). Using the intact hand for objective assessment of phantom hand-perception. *European Journal of Pain*.

[35] Peltz E, Seifert F, Lanz S, Müller R, Maihöfner C (2011). Impaired hand size estimation in CRPS. *Journal of Pain*.

[36] Gieteling EW, van Rijn MA, de Jong BM (2008). Cerebral activation during motor imagery in complex regional pain syndrome type 1 with dystonia. *Pain*.

[37] Murphy MJ (1991). Effects of cervical traction on muscle activity. *Journal of Orthopaedic and Sports Physical Therapy*.

[38] Yue G, Cole KJ (1992). Strength increases from the motor program: comparison of training with maximal voluntary and imagined muscle contractions. *Journal of Neurophysiology*.

[39] Fansler CL, Poff CL, Shepard KF (1985). Effects of mental practice on balance in elderly women. *Physical Therapy*.

[40] Maring JR (1990). Effects of mental practice on rate of skill acquisition. *Physical Therapy*.

[41] Zimmermann-Schlatter A, Schuster C, Puhan MA, Siekierka E, Steurer J (2008). Efficacy of motor imagery in post-stroke rehabilitation: a systematic review. *Journal of NeuroEngineering and Rehabilitation*.

[42] Dickstein R, Dunsky A, Marcovitz E (2004). Motor imagery for gait rehabilitation in post-stroke hemiparesis. *Physical Therapy*.

[43] Vlaeyen JWS, Linton SJ (2000). Fear-avoidance and its consequences in chronic musculoskeletal pain: a state of the art. *Pain*.

